# Use of rapid diagnostic tests for the detection of ancient malaria infections in dental pulp from the sixth century in Versailles, France

**DOI:** 10.1186/s12936-023-04582-7

**Published:** 2023-05-09

**Authors:** Mahmoud A. Boualam, Annick Heitzmann, Florence Mousset, Gérard Aboudharam, Michel Drancourt, Bruno Pradines

**Affiliations:** 1 IHU Méditerranée Infection, 19–21 Bd Jean Moulin, 13005 Marseille, France; 2 Aix-Marseille Univ, IRD, MEPHI, AP-HM, 19–21 Bd Jean Moulin, 13005 Marseille, France; 3 Direction du Patrimoine et des Jardins, Château de Versailles, Place d’Armes, 78008 Versailles, France; 4 Direction régionale des affaires culturelles d’Île-de-France, Service Régional de l’archéologie, 311 Rue Le Peletier, 75009 Paris, France; 5 Ecole de Médecine Dentaire, Aix-Marseille Univ, Bd Jean Moulin, 13005 Marseille, France; 6 Unité parasitologie et entomologie, Département microbiologie et maladies infectieuses, Institut de recherche biomédicale des armées, 19–21 Bd Jean Moulin, 13005 Marseille, France; 7 Aix-Marseille Univ, IRD, SSA, AP-HM, VITROME, 19–21 Bd Jean Moulin, 13005 Marseille, France; 8 Centre national de référence du paludisme, 19–21 Bd Jean Moulin, 13005 Marseille, France

**Keywords:** Paleomicrobiology, Ancient malaria, *Plasmodium vivax*, *Plasmodium falciparum*, Europe, Co-infection, Immunodetection

## Abstract

**Background:**

Paleomicrobiological data have clarified that *Plasmodium* spp. was circulating in the past in southern European populations, which are now devoid of malaria. The aim of this study was to evaluate the efficacy of immunodetection and, more particularly, rapid diagnostic tests (RDT), in order to further assess *Plasmodium* infections in ancient northern European populations.

**Methods:**

A commercially available RDT, PALUTOP^®^ + 4 OPTIMA, which is routinely used to detect malaria, was used to detect *Plasmodium* antigens from proteins recovered from ancient specimens extracted from 39 dental pulp samples. These samples were collected from 39 individuals who were buried in the sixth century, near the site of the current Palace of Versailles in France. Positive and negative controls were also used. Antigens detected were quantified using chemiluminescence imaging system analysis.

**Results:**

*Plasmodium* antigens were detected in 14/39 (35.9%) individuals, including *Plasmodium vivax* antigens in 11 individuals and *Plasmodium falciparum* antigens co-detected in two individuals, while Pan-*Plasmodium* antigens were detected in three individuals. Controls all yielded expected results.

**Conclusions:**

The data reported here showed that RDTs are a suitable tool for detecting *Plasmodium* spp. antigens in ancient dental pulp samples, and demonstrated the existence of malaria in Versailles, France, in the sixth century. *Plasmodium vivax,* which is regarded as being responsible for an attenuated form of malaria and less deadly forms, was the most prevalent species. This illustrates, for the first time in ancient populations, co-infection with *P. falciparum*, bringing into question the climate-driven ecosystems prevailing at that time in the Versailles area.

## Background

Malaria is a deadly infection and a public health concern. In 2021, there were an estimated 247 million cases worldwide, resulting in 619000 deaths [1]. Deadly malaria, however, is far from being evenly distributed around the world, with about 96% of malaria deaths globally occurring in 29 countries. In 2021, four countries accounted for just over half of all malaria deaths globally, namely Nigeria (31%), the Democratic Republic of the Congo (13%), Niger (4%) and the United Republic of Tanzania (4%). The disease is caused by *Plasmodium* parasites and is transmitted by the bite of infected female *Anopheles* mosquitoes [2]. Six of the estimated 250 malaria species are recognized as human pathogens, including *Plasmodium falciparum*, *Plasmodium vivax*, *Plasmodium ovale wallikeri*, *P. ovale curtisi, Plasmodium malariae*, and *Plasmodium knowlesi.* Malaria is no longer endemic in many developed countries and was eliminated from most Western European countries between the 1930s and 1970s. However, the disease continues to represent a challenge in terms of prevention, diagnosis, and patient management. Malaria is imported into Europe by travellers and migrants from endemic areas, as well as by visitors from endemic areas and armed forces personnel returning home after overseas operations. Among developed countries, mainland France reports the greatest number of cases of imported malaria, with 4,995 estimated cases in 2021 (B Pradines, personal data, Centre National de Référence du Paludisme). The last proven autochthonous case in France was detected in 2006 in Corsica [3] and the last outbreak of locally acquired malaria infection (*P. vivax*) in Europe occurred in 2011–2012 in Greece [4].

**Fig. 1:**
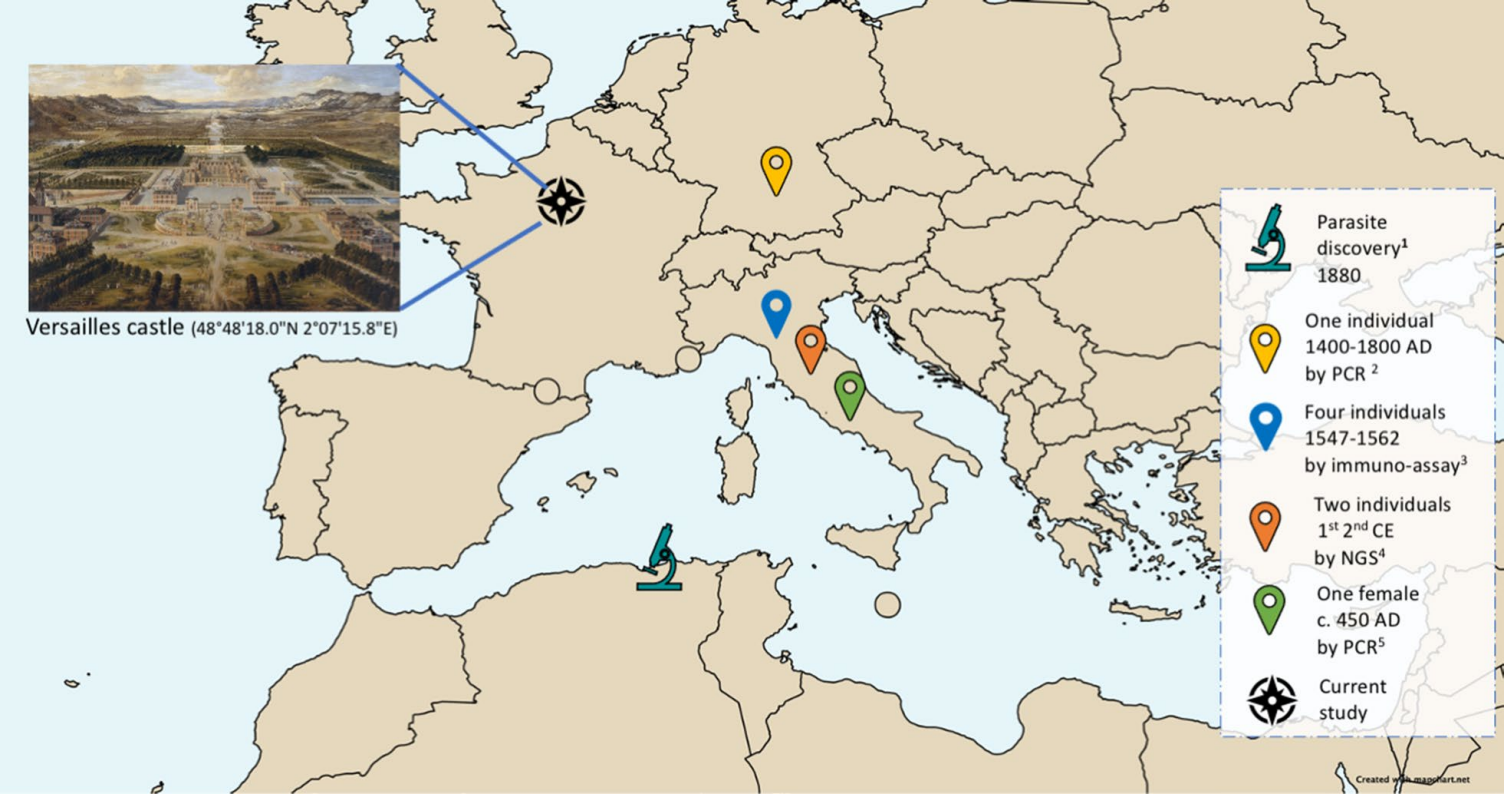
European studies detecting the presence of ancient malaria. 1: First parasite discovery by Laveran [48], 2: Parasite detection in bone by PCR [17], 3: Parasite detection in bone by immune assay [18], 4: Parasite detection in molar by NGS [36] 5: Parasite detection in bone by PCR [35]

Paleomicrobiological and paleogenetic data, reinforced by ancient textual and iconographic descriptions, indicate previous occurrences of malaria in Europe [5]. However, the antiquity of malaria in human populations is poorly understood and has previously focussed on human remains from ancient Egypt, including mummies and non-mummy samples such as bones and teeth [6–16]. Studies have also been carried out in Italy [17–21] using molecular detection assays, including immunoenzymatic, polymerase chain reaction (PCR), next generation sequencing (NGS) and indirect immunofluorescence assays. The aim of this study was to evaluate the use of rapid diagnostic tests (RDT) to detect *Plasmodium* antigens in dental pulp from teeth collected from human remains. Here, immunochromatography detection of *Plasmodium* specific antigens in the presence of the appropriate controls was assessed on human remains collected from a sixth century Merovingian necropolis near the site of the world-renowned Palace of Versailles (Fig. 1).

## Methods

### Immunochromatographic assay

The malaria RDT used in the study was PALUTOP^®^ + 4 OPTIMA (Biosynex, Illkirch-Graffenstaden, France), which detects three antigens of human malaria, Pf HRP-2 (histidine rich protein-2) specific to *P. falciparum*, pLDH (lactate dehydrogenase) specific to *P. vivax,* and Pan-pLDH common to all *Plasmodium* species (pan species). Test strips were extracted for quantitative reading using the FUSION FX chemiluminescent imaging system and the ImageQuant TL software (Witec AG, Sursee, Switzerland). The possible result scenarios described by the supplier are the following: the test is considered as invalid if the internal control band remains negative. In the presence of a positive internal control band, the test result is considered as negative if all the other bands are negative. In the presence of a positive internal control band, the sample is considered as containing specific antigen of *P. falciparum*, suggesting current infection, if the Pan (Pan *Plasmodium*-pLDH) and *Pf* (*P. falciparum* HRP-2) bands are positive. The presence of a positive internal control band with only a positive *Pf* band is predictive of a *P. falciparum* current infection or effective post-treatment: HRP2 proteins can be found up to 45 days after effective treatment. The presence of a positive internal control band with positive *Pv* (*P. vivax* pLDH) and Pan bands suggests a vivax infection. The presence of a positive internal control band with only the Pan band is predictive of infections with *P. ovale*, *P. malariae* or *P. knowlesi*. If all the bands are positive, this result suggests the presence of a mixed infection with at least *P. falciparum* and *P. vivax*.

### Malaria controls

To validate the assay, three fresh negative blood samples, previously tested by a routine malaria test (Alethia^®^ Malaria LAMP assay, Meridian Bioscience, Cincinnati, USA), were taken after validation of the approach by the laboratory ethics committee. Additionally, five samples infected with different *Plasmodium* species at different parasitaemia were used as control (three samples with *P. falciparum* parasites at 16%, 1.6% and 0.01%, one sample with *P. vivax* parasites at < 0.01% and one sample with *P. ovale* parasites at < 0.01%). Moreover, three *P. falciparum* parasites collected from in vitro cultures were used (parasitaemia at 0.1%, 1.6% and 3%, respectively). These *P. falciparum* parasites were previously maintained in human red blood cells in RPMI 1640 medium (Invitrogen, Paisley, UK) supplemented with 10% human serum (Etablissement Français du Sang, Marseille, France) and buffered with 25 mM HEPES and 25 mM NaHCO_3_ under controlled atmospheric conditions that consisted of 10% O_2_, 5% CO_2_ and 85% N_2_ with a humidity of 95%.

All these samples (including the negative controls) were stored at room temperature (average 21 °C) for five days in ambient air (average humidity, 15%) to mimic ancient blood specimens. Both fresh and derived mock-ancient blood specimens were then tested by routine laboratory diagnostic methods, the Alethia® Malaria LAMP assay (Meridian Bioscience, Cincinnati, USA), and immunochromatographic assay (PALUTOP^®^ + 4 OPTIMA, Biosynex, Illkirch-Graffenstaden, France).

### Anthropological sites

During an archaeological excavation carried out in 2013 in the courtyard of the Grand Commun of the Palace of Versailles (48° 48′ 18.0′′ N, 2° 07′ 15.8′′ E), part of a Merovingian necropolis dating from the sixth century was discovered. Dating was carried out using the C14 dating method and was applied to two tombs and funeral materials consisting of pearls, ceramics and metal artefacts. In total, 35 graves contained objects, including some 284 beads and 127 metal remains. Of the human remains of 109 people which were excavated, 13 were women, 15 were men and 81 were of undetermined sex, aged between one and approximately 50 years old. Thirty-nine of these individuals had at least one intact tooth available for study. This comprised 12 men, 10 women and 17 individuals of undetermined sex (Table 1). Bones recovered from the burial site were extremely damaged (missing extremities, low number of so-called “short” bones such as ribs, vertebrae, and pelvises), but teeth were well preserved. As regards dental health information, the individuals exhibited dental wear, dental calculus, cavities and some abscesses. Anthropological examination also indicated post-cranial enthesopathy in two individuals and arthritic lesions in three individuals. Signs of infectious pathology were also observed in the bones [22]. Malaria does not cause any pathognomonic lesions in the skeleton and the bones were too damaged to conclude if porous skeletal lesions potentially associated with anaemia were due to malaria.

**Table 1:** Characteristics of the 39 samples and results of the detection by the PALUTOP^®^ + 4 OPTIMA RDT

Laboratory ID	Estimated age	Sex	Pan-pLDH	PvLDH	PfHRP2
Os34	Young adult	M	** + **	−	−
Os38	Older adult	UD	** + **	** + **	−
Os50	Young adult	M	−	−	−
Os66	Mature adult	UD	** + **	** + **	−
Os97	Young adult	F	** + **	** + **	** + **
Os125	Adult	M	−	−	−
Os131	Adult	M	** + **	** + **	−
Os137	Adult	M	−	−	−
Os174	Young adult	M	−	−	−
Os178	Mature adult	M	−	−	−
Os182	Young adult	UD	** + **	**-**	−
Os184	Young adult	F	−	−	−
Os199	Mature adult	M	−	−	−
Os219	UD	UD	−	−	−
Os223	Older adult	UD	** + **	** + **	−
Os232	Adult	M	** + **	** + **	−
Os237	UD	UD	−	−	−
Os243	Adult	M	** + **	** + **	** + **
Os263	Mature adult	UD	−	−	−
Os282	Young adult	UD	** + **	−	−
Os283	Young adult	UD	−	−	−
Os286	Adult	UD	−	−	−
Os297	Adult	F	−	−	−
Os319	Adult	UD	** + **	** + **	−
Os323	Young adult	UD	−	−	−
Os325	Older adult	UD	−	−	−
Os334	Adult	F	−	−	−
Os337	Adult	F	−	−	−
Os338	Adult	F	−	−	−
Os339	Adult	F	−	−	−
Os340	Adult	F	−	−	−
Os371	Adult	F	−	−	−
Os377	Young adult	UD	−	−	−
Os380	UD	UD	** + **	** + **	−
Os398	Adult	M	−	−	−
Os399	Adult	M	** + **	** + **	−
Os411	Adult	F	−	−	−
Os429	Mature adult	UD	** + **	** + **	−
Os431	Mature adult	UD	−	−	−

*M* male, *F* female, *U * undetermined

### Sample preparation

**Fig. 2:**
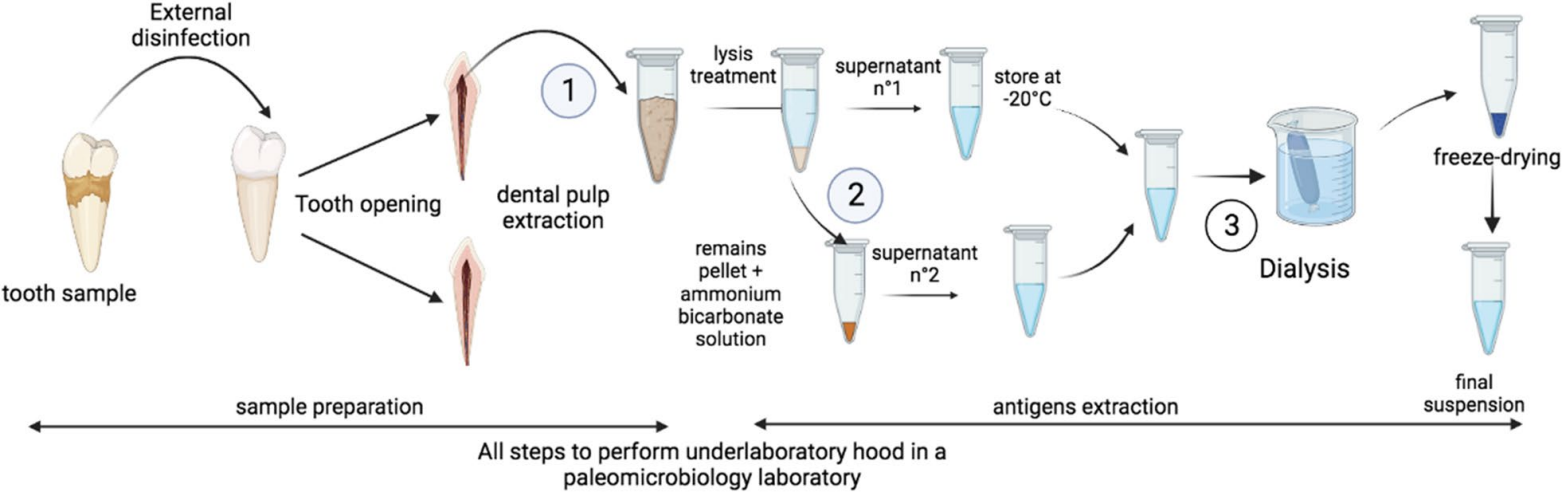
Working approach for the screening of the site of Versailles in the sixth century

Dental pulp, which is known to contain blood at the time of an individual’s death, was used as an anthropological material suitable for the detection of blood-borne *Plasmodium* parasites [23]. For each one of the 39 intact teeth collected from 39 individuals, the outer surface of the tooth was disinfected using sterile gauze soaked in 100% ethanol followed by 9% bleach. The dental pulp was then extracted, as previously described [24]. In brief, a sterile diamond disc was used to create a fracture line along the length of the tooth without reaching the pulp cavity. The tooth was then fractured into two parts using a sterile straight osteotome. Using a dental excavator, the remaining dental pulp was scraped into a sterile Eppendorf tube. The teeth were placed under a hood for pre-opening, fracture and pulp extirpation to avoid external contamination. The selected teeth were divided into four batches for pulp extirpation, with both half-pulps being stored in an Eppendorf tube (Fig. 2).

### Prevention of contamination

To avoid any risk of contamination, all manipulations were carried out in a laboratory entirely dedicated to paleomicrobiology work. All the instruments necessary for opening the teeth were also disinfected in the same way before each tooth was handled. After each batch, the handling instruments were autoclaved. To control contamination, one extraction control consisting of an extraction blank was added to each extraction batch.

### Ancient antigen extraction

Pulp samples were treated in the same way for protein extraction, as previously reported [25], firstly by adding 200 μL of ethylenediaminetetraacetic acid (EDTA) and sonicated 5 × 1 min at 6 000 g and incubated overnight at 4 °C under agitation. The following day, samples were centrifuged for 30 min at 17 000 g at room temperature and the supernatant (referred to as supernatant no 1) was stored at − 20 °C. The remaining pellets were washed twice with 200 μL of sterile distilled water before being suspended in 100 μL solution of 50 mM ammonium bicarbonate solution (pH 7.4) and incubated at 75 °C for 48 h. Supernatant no 2 was then collected by centrifugation at 17 000 g for 30 min at room temperature, and stored at − 20 °C. The two supernatants (no 1 and no 2) were pooled and dialysed first using Slide-A-Lyzer MINI Dialysis Device, 2 K MWCO (Pierce Biotechnology, Rockford, IL, USA) with 1 L of dialysis solution (50 mM ammonium bicarbonate) for 4 h, and then overnight. The resulting solution contained proteins recovered from ancient specimens which were freeze-dried and suspended in 30 μL of PBS. A Qubit assay was performed for protein quantification for each extracted sample.

## Results

The three fresh *Plasmodium*-negative blood samples used as negative controls, as well as the protein extract from negative mock-ancient blood were negative. The results of the *P. falciparum* cultures, with 0.1%, 1.6% and 3% parasitaemia, respectively, were positive for the Pan-pLDH antigen and the *P. falciparum* HPR2 antigen, as well as the protein extractions carried out on imitated ancient *P. falciparum* collected from in vitro cultures (Table 2). These results validated the immunochromatographic assay. The *P. falciparum* isolates with 0.01%, 1.6% and 16% parasitaemia, respectively, were positive for the Pan-pLDH antigen and the *P. falciparum* HPR2 antigen, as well as protein extractions carried out after blood dehydration. For the *P. vivax* sample with < 0.01% parasitaemia, the detection was positive for the Pan-pLDH antigen but not for the *P. vivax* pLDH antigen, most likely due to the low parasitaemia. It should be noted that the protein extraction from dried blood was positive, with low intensity for both Pan-pLDH antigens and for the *P. vivax* pLDH antigens. The *P. ovale* sample, with < 0.01% parasitaemia, as well as the protein extraction were negative for all test antigens, including the Pan-pLDH antigen, most likely due to the low sample parasitaemia and low specificity of the test for this species [26].

**Table 2:** Results of the detection of *Plasmodium* by the PALUTOP^®^ + 4 OPTIMA RDT in the control samples

Species	Parasitaemia (%)	Pan-pLDH	PvLDH	PfHRP2
Negative	0.00	−	−	−
Negative	0.00	−	−	−
Negative	0.00	−	−	−
*P. falciparum*	0.1	+	−	+
*P. falciparum*	1.6	+	−	+
*P. falciparum*	3.0	+	−	+
*P. falciparum*	0.01	+	−	+
*P. falciparum*	1.6	+	−	+
*P. falciparum*	16.0	+	−	+
*P. vivax*	< 0.01	+	−	−
*P. ovale*	< 0.01	−	−	−

**Fig. 3:**
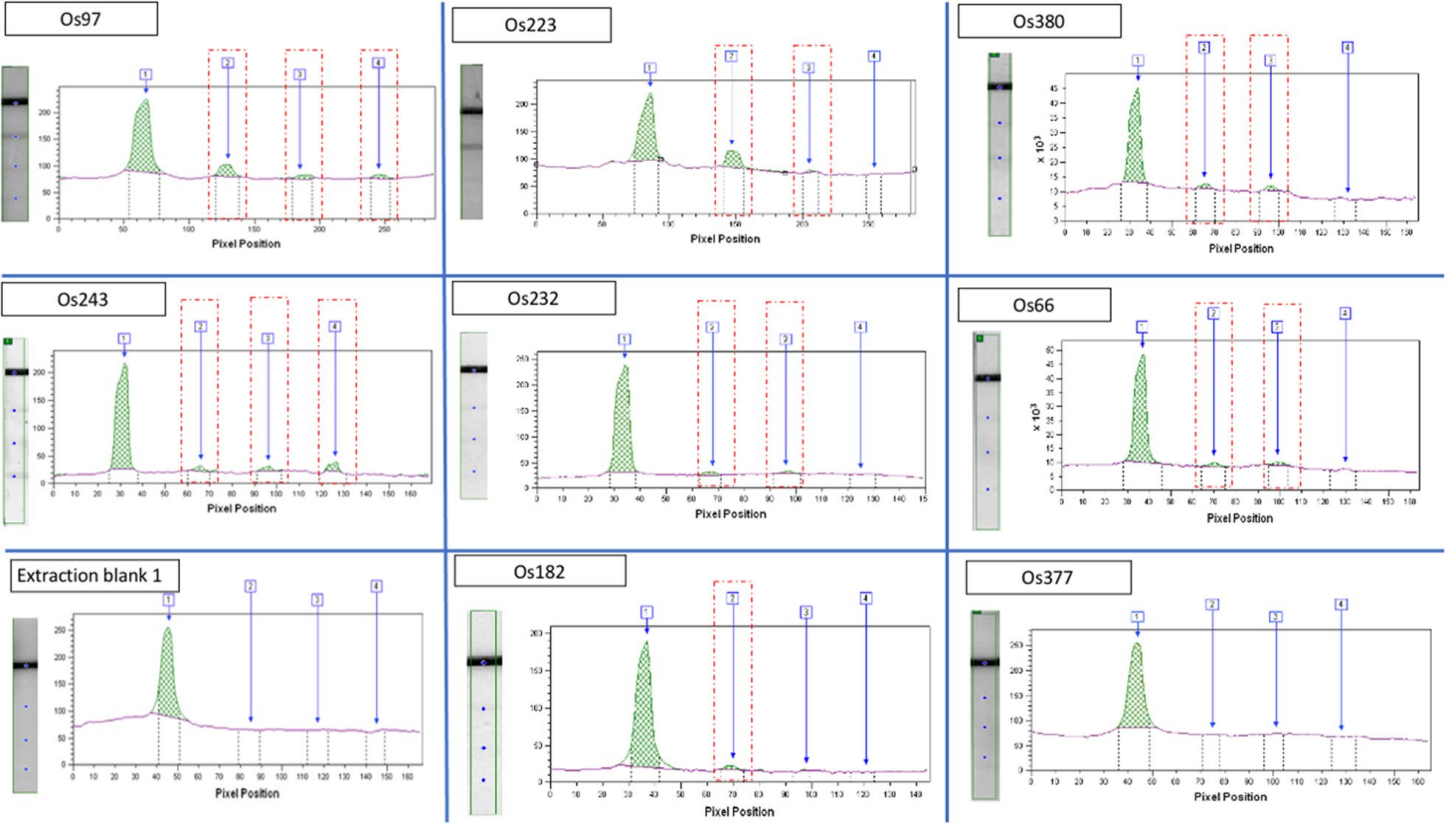
Quantitative analysis of bands extracted from PALUTOP^®^ + 4 OPTIMA, imaging and reading using the FUSION FX, band1: internal control; Band2: Pan-pLDH malaria pan species antigen; band 3: PvLDH *P. vivax* antigen; band 4: PfHRP2 *P. falciparum* antigen

As for the ancient specimens, the presence of *Plasmodium* antigens was detected in 14/39 (35.9%) individuals (Table 1, Fig. 3). The five negative controls were negative. Three samples only detected the Pan-pLDH antigen (Os34, Os182 and Os282), nine detected both the Pan-pLDH and PvLDH antigens (Os38, Os66, Os131, Os223, Os232, Os319, Os380, Os399 and Os429), and two detected the three antigens (Pan-pLDH, PvLDH and PfHRP2) (Os97 and Os243). All control curves appeared with the negative controls and no curve corresponding to a malarial antigen appeared after the analysis.

Although the size of the tested population was limited to 39 individuals, a prevalence of malaria-infected individuals of 35.9% was found, suggesting a relatively high prevalence of malaria infection in this area of Versailles, even when the sensitivity of the test used in this study was taken into account. Indeed, PALUTOP® + 4 OPTIMA performance tests demonstrated a sensitivity interval of 91–98.7% for *P. falciparum* infection, and 82.9–100% for *P. vivax* infection [27–29]. In our study, three samples yielded only the pan-*Plasmodium* Pan-pLDH antigen but not the least sensitive antigens for *P. falciparum* and *P. vivax* detection in comparison with PfHRP2 and PvLDH [29], suggesting these three individuals may have been infected with *P. ovale* and/or *P. malariae* or with low density *P. vivax* infections.

## Discussion

The results reported here, which used an immunochromatographic assay in the presence of appropriate controls, detected the presence of *Plasmodium* spp. antigens in the sixth century remains excavated from a site near the current Palace of Versailles in northern France. In this study, dental pulp, which has been recognized as containing blood at the time of death [30], proved to be suitable for the immunochromatographic detection of *Plasmodium* antigens. This is the first time that immunochromatographic tests and, more particularly, RDTs have been used to detect *Plasmodium* spp*.* in dental pulp. It is also the first time that the biological presence of old malaria infections has been detected in France.

The preservation of protein samples over time and their stability have been demonstrated by several published studies, as resistance of proteins to degradation is high and greater than that of DNA, at least in ancient human remains [14–16, 31–33]. This approach is interesting for several reasons. Studies have already been carried out based on the detection of ancient antigens using immunochromatographic techniques for the diagnosis of cases of ancient malaria, in particular on samples collected from mummies [5]. In Italy, two studies diagnosed *P. falciparum* infection, the first from bone samples dating from the sixteenth century from the Medici family of Florence [18], and another in Sardinia on 34 individuals dating from between the fourteenth century BCE and the sixteenth century CE [34]. Investigations of bone samples from an infant cemetery in Lugnano, dating from 450 CE, yielded positive PCR results for *P. falciparum* in two samples belonging to the same individual [35]. *Plasmodium falciparum* mtDNA fragments were also detected by NGS in two individuals dating from the first and second centuries CE in southern Italy after hybridisation capture [36].

RDT sensitivity is generally greater than 90% and specificity is greater than 80% [37]. However, the limitation of the tests consists in the possibility of false positives, for example in the presence of a rheumatoid factor, especially with the first generation of RDT. The substitution of IgG by IgM in the novel generation of tests has considerably reduced cross-reactivity [15, 37, 38]. False negative results have been observed in the case of very low parasitaemia (< 100 parasites/µL) [37], as well as in the situation of a deleted or a mutated *Pfhrp2* gene [38]. The comparative study of malaria diagnostic techniques in ancient materials has shown that positive immunoassays correlated with detection of *Plasmodium* spp. NGS reads, confirming the usability of immunoassays on ancient samples with preserved specificity and sensitivity similar to that achieved with modern samples [15].

Most individuals were found to be positive for the *P. vivax* antigen, an observation which is in line with previous observations of *P. vivax* in three individual samples collected between 1942 and 1944 in the Ebro Delta in Spain, before eradication, and investigated by metagenomics 70 years later [39, 40]. Further climate reconstitution of the Middle Ages suggests climatic conditions which were compatible with the survival of *P. vivax* [41, 42]. One study mentioned that current lineages of *P. vivax* may have a South Asian origin, by modelling studies based on climatic variations [43]. These indicated a potential summer generation of *P. vivax*-like malarial protozoan, with a favourable climate for mosquito development, which probably covered western and central parts of Europe, where climatic conditions were mild. These results support the continued survival of *P. vivax*-like parasites throughout the quaternary era in Europe [44]. The observation of *P. falciparum* and *P. vivax* co-infection currently occurring in between 5 and 7% of all malaria cases [45] may further indicate a probable co-circulation of several *Anopheles* mosquitoes at that time, in the absence of any retrieved published data on insects in the mediaeval period. However, some historical sources reported marshland in this part of France [46], biotopes which are associated with the *Anopheles* mosquitoes. Moreover, *Anopheles maculipennis*, *Anopheles atroparvus*, *Anopheles sacharovi* and *Anopheles labranchiae*, all known to be possible vectors for *P. falciparum* and *P. vivax* [47], have been historically documented in the Merovingian period in Europe [21]. Taken together, the historical and paleomicrobiological data reported here converge on the verisimilitude of malaria existing in this region of France during the mediaeval period.

There is, however, a lack of anthropological data to further assess the morbidity of *Plasmodium* spp. in these individuals. In modern times, the detection of *Plasmodium* spp. in the blood is associated with a wide spectrum of medical conditions, from asymptomatic carriage in apparently healthy individuals to death.

## Conclusions

This is the first time that the *Plasmodium* antigens captured from ancient specimens by RDTs, have been quantified, especially using chemiluminescence imaging system analysis. Immunochromatographic tests and, more particularly, RDTs were showed to be a suitable tool for detecting *Plasmodium* spp. antigens in ancient dental pulp. Immunodetection proved to be a fast, sensitive and very useful technique for screening ancient samples for infectious disease, for the purposes of carrying out an initial diagnosis directly on the excavation sites. However, the lack of validation of DNA-based detection of *Plasmodium* is a limit of this study.

Secondly, this is the first biological detection of malaria at a time of transition between the Roman Empire and the Merovingian reign, within the territory of modern-day France. The study of other sites, in particular sites that used to be marshy, would be of great interest in terms of gaining a better understanding the determinants of the current eradication of malaria in Europe.

## Data Availability

The datasets analysed in this study are available from the corresponding author on reasonable request.
